# Emerging Roles of the Atypical Chemokine Receptor 3 (ACKR3) in Cardiovascular Diseases

**DOI:** 10.3389/fendo.2022.906586

**Published:** 2022-06-29

**Authors:** Vincent Duval, Paul Alayrac, Jean-Sébastien Silvestre, Angélique Levoye

**Affiliations:** ^1^ Université Paris Cité, Institut National de la Santé Et Recherche Médicale (INSERM), Paris Cardiovascular Research Center PARCC, Paris, France; ^2^ UFR Santé Médecine Biologie Humaine, Université Sorbonne Paris Nord, Bobigny, France

**Keywords:** atypical chemokine receptors, ACKR3, chemokine, cardiovascular diseases, signalling

## Abstract

Chemokines, and their receptors play a crucial role in the pathophysiology of cardiovascular diseases (CVD). Chemokines classically mediate their effects by binding to G-protein-coupled receptors. The discovery that chemokines can also bind to atypical chemokine receptors (ACKRs) and initiate alternative signaling pathways has changed the paradigm regarding chemokine-related functions. Among these ACKRs, several studies have highlighted the exclusive role of ACKR3, previously known as C-X-C chemokine receptor type 7 (CXCR7), in CVD. Indeed, ACKR3 exert atheroprotective, cardioprotective and anti-thrombotic effects through a wide range of cells including endothelial cells, platelets, inflammatory cells, fibroblasts, vascular smooth muscle cells and cardiomyocytes. ACKR3 functions as a scavenger receptor notably for the pleiotropic chemokine CXCL12, but also as a activator of different pathways such as β-arrestin-mediated signaling or modulator of CXCR4 signaling through the formation of ACKR3-CXCR4 heterodimers. Hence, a better understanding of the precise roles of ACKR3 may pave the way towards the development of novel and improved therapeutic strategies for CVD. Here, we summarize the structural determinant characteristic of ACKR3, the molecules targeting this receptor and signaling pathways modulated by ACKR3. Finally, we present and discuss recent findings regarding the role of ACKR3 in CVD.

## Introduction

Cardiovascular diseases (CVD) are the leading cause of death worldwide. CVD cover all heart and blood vessel dysfunctions, including hypertension, coronary artery disease, cerebrovascular disease (stroke or transient ischemic attack), myocardial infarction (MI), heart failure and peripheral vascular diseases such as aneurysms. The main pathology behind CVD is atherosclerosis, a chronic inflammatory disease of the arterial wall characterized by the development of lesions caused by increasing endothelial permeability and the accumulation of low density lipids in the intima (1, 2). Subsequent lipid modifications trigger endothelial dysfunction, migration and recruitment of inflammatory cells through the action of several adhesion molecules and chemokines. Monocyte-derived macrophages take up these lipids, resulting in the formation of foam cells (3). Smooth muscle cells migrate from the media to the intima, where they proliferate and extracellular matrix molecules such as collagen and proteoglycans are synthesized, leading to the creation of a fibrous cap. This process promotes plaque development within the arterial wall resulting mainly to vascular occlusion through thrombus formation and subsequent tissue ischemia with severe clinical consequences, such as stroke or MI.

Treatment of risk factors, development of potent anticoagulant and antiplatelet therapies, and the widespread availability of revascularization methods have significantly improved the treatment and prevention of CVD. However, according to the WHO, approximately 17.9 million deaths per year are caused by CVD. In 2030, this number expected to rise to more than 23.6 million deaths (4). This clearly supports the necessity to better understand the cellular and molecular mechanisms regulating CVD development, such as inflammatory response after injury or during atherosclerosis in order to propose novel therapeutic strategies. Chemokines and their receptors play a key role in these processes and thereby represent promising potential therapeutic targets in the treatment of CVD. Several reviews have already highlighted the importance of the Atypical Chemokine Receptors (ACKRs) in CVD (5). The aims of this mini-review are to focus on the specific roles of a ACKR subtype i.e., ACKR3, in CVD, to detail the mechanisms leading to the modulation of the ACKR3-related functions, and to discuss the therapeutic opportunities based on targeting ACKR3.

## Atypical Chemokine Receptors

Chemokines are small cytokines (8-12 KDa) that trigger cell chemotaxis along a concentration gradient. To date, chemokines have been classified into 4 families based on the position of the first two cysteine residues (CC, CXC, CX3C, XC). Chemokines can be expressed by different cell types such as endothelial cells, fibroblasts, cardiomyocytes but also inflammatory cells. They can be detected within injury tissues, where they regulate the recruitment of leukocytes to sites of inflammation and control tissue homeostasis through their ability to shape resident cell-related functions (6). Indeed, chemokines can also modulate, through their binding to multiple receptors, a plethora of other cellular functions such as proliferation, survival, differentiation and cytokine release. Conventional (cCKRs) and atypical (ACKRs) comprise the two types of chemokine receptors (7). Most chemokines are recognized by a set of cCKRs belonging to the G-protein-coupled receptors (GPCRs) family associated with G-protein coupling and β arrestin activation leading to functional redundancy within the chemokine system. Currently, they are 23 cCKRs classified according to the type of chemokine they bind. However, the complexity of the chemokine system is increased by the presence of ACKR subtypes (8). ACKRs are seven transmembrane domain receptors structurally homologous to GPCRs. Currently, the ACKR family comprises five major receptors: ACKR1/DARC (Duffy Antigen Receptor for Chemokines), ACKR2/D6, ACKR3/CXCR7, ACKR4/CCRL1 (CC-Chemokine Receptors like 1) and ACKR5/CCRL2 (9). ACKRs can bind to a variety of different ligands to elicit their biological effects. Originally, ACKRs have been characterized as decoy or silent receptors because they fail to induce a classical signalling signature characteristic for GPCRs in response to the binding of the chemokine. The term “Atypical” emerges from the observation that ACKRs have either lacked or modified canonical DRYLAIV motif located at the end of the third transmembrane domain and within the second intracellular loop, essential for binding and activation of G-proteins. Their failure to couple to G-proteins and to induce “classical” GPCR signalling led to their exclusion from the chemokine receptor nomenclature and to their initial designation as “silent” receptor. However, modifications of the DRYLAIV motif present in the CKR XCR1 and CXCR6 both known to signal *via* PTX-sensitive Gαi proteins have challenged this dogma. This has confirmed that modifications of the DRYLAIV motif were neither a indicator neither the only determinant for the lack of G-protein coupling (10, 11). Hence, ACKRs are atypical related to their ability to activate intracellular pathways through G-protein-independent signalling (12). ACKRs were initially considered as scavenger receptors by their ability to promote the targeting and the degradation of the bound chemokine in lysosomes. The typical D6 receptor is a prototypical example of receptors harbouring scavenging function (13). The transcytosis function allowing the transport of chemokines across biological barriers has also been proposed for some ACKR such as DARC (14). Thus, ACKRs appear as major regulators of chemokine internalization, degradation, and transcytosis (15). These activities may lead to the establishment of chemokine concentration gradient within tissue which may regulate the bioavailability of a defined chemokine and subsequently the cCKR-dependent signalling (16). In addition, ACKRs may also modulate the responses to chemokines through receptor heterodimerization (17) or by sustaining their own signalling pathways.

## ACKR3 and its Ligands

ACKR3, initially thought to be an orphan 7 transmembrane domain receptor, has been identified in dog in 1989 (18), human in 1991 (19) and was named RDC1. The murine form was identified in 1998 (20) and showed more than 85% homology with the human and canine RDC1. ACKR3 losts its orphan receptor status when CXCL12 initialy named SDF-1 (Stromal Derived Factor-1) was discovered as one of its ligands (21). CXCL12 mainly acts through its classical CXCR4 receptor (22, 23). Three and six CXCL12 isoforms have been identified in mouse and human, respectively (24). Although, all CXCL12 isoforms are likely able to bind to ACKR3 their specific roles in ACKR3-related functions remain to be defined. However, with a 10-fold higher affinity than CXCR4, RDC1 is able to induce chemotaxis of lymphocytes in response to the CXCL12α isoform (21, 25). As a consequence, RDC1 was then considered to belong to the CXC chemokine receptor family and was renamed CXCR7. Unlike cCKRs, CXCR7 is not always associated with Gαi proteins and does not induce calcium mobilization. Its canonical DRYLAIV motif known as essential for G protein coupling is altered. This structural difference has led to a change in its classification from CXCR7 to ACKR3 (26). However, it should be noted that the inability of ACKR3 to couple heterotrimeric Gα proteins remains debated. Indeed, ACKR3 is able to interact with Gαi1 and Gαi3 proteins in HEK293 cells (27, 28) but not trigger a calcium response. Only, primary rodent astrocytes and human glioma cells respond to CXCL12-dependent activation of ACKR3 with increases in intracellular calcium which can be reversed by pertussis toxin (27, 28) (**Table 1**). Other chemokines, CXCL11 (or I-TAC, interferon-inducible T-cell alpha chemoattractant), a CXCR3 agonist, has also been identified as a ligand of ACKR3 (21). Recently, vCCL2, also known as vMIPII, a viral chemokine, was found to act as a partial agonist of ACKR3 which can induce β-arrestin recruitment and Mitogen Activated Protein Kinases (MAPK) activation (44). Additional non-chemokine ligands have also been identified including the macrophage migration inhibitory factor (MIF) (29, 45), adrenomedullin, pro-adrenomedullin N-terminal 20 Peptides and its processed form PAMP12 (30, 46) as well as several opioid-derived peptides (30, 31, 47). Furthermore, several synthetic ligands for ACKR3 were synthesized, such as the cyclic peptide agonist TC14012 derived from T140 (32), VUF11207 as well as VUF11403 (33), ACT-1004-1239 (34) and CCX771 (35). AMD3100, a well-known CXCR4 antagonist, has been identified as a allosteric modulator of ACKR3 but only at high concentrations (48) (**Table 1**).

**Table 1:** ACKR3: ligands, signalling and functions.

**Ligands**	**References**
Natural: CXCL12, CXCL11, PAMP-12, MIF, opioïd peptides Synthetic: TC14012, VUF11207, VUF11403, CCX771, ACT-1004-1239	(21, 25, 29, 30) (31–35)
**Interaction partners**	
CXCR4 Connexin 43	(17–36) (28)
**Signalling pathways**	
β-arrestin Gαi-protein	(16, 32, 35) (28, 37)
**Homeostasic functions**	
Development of cardiovascular system Vasculogenesis Neuronal migration	(38–40) (41) (42, 43)

## ACKR3 Signalling Pathways

**Figure 1:**
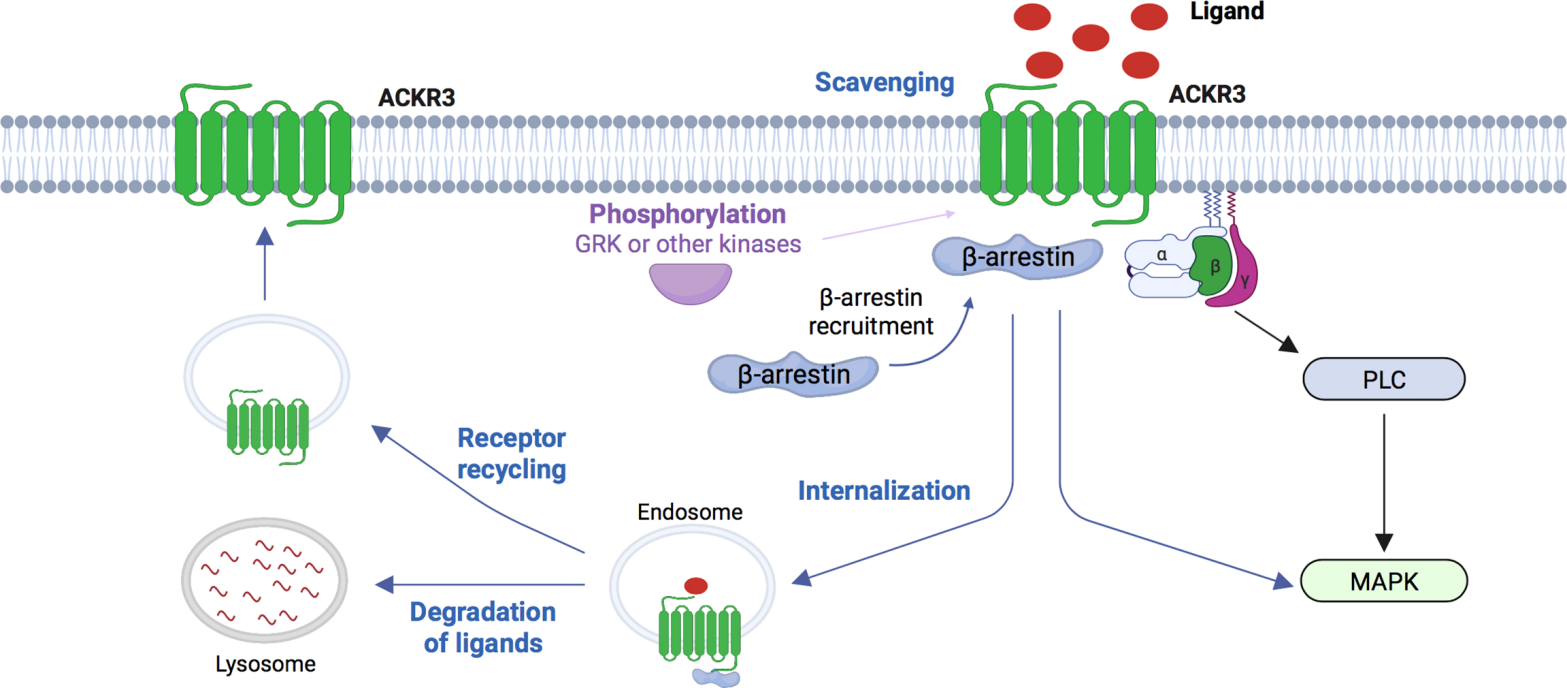
Schematic representation of signaling pathways for ACKR3. Chemokines ligands CXCL12 and CXCL11 can induce phosphorylation of ACKR3 by GRK or others kinases, the recruitment of β-arrestins and trigger intracellular G-protein independent signaling. ACKR3 is also able to scavenge various ligands (CXCL12, CXCL11, ADM, PAMP, PAMP12, opioїd peptides) at the cell surface, internalized these ligands which subsequently result in degradation in lysosome while the receptor recycles to the cell membrame. ACKR3 is also able to interact with G-proteins without to lead to their activation. However, in two specific cellular types, primary rodent astrocytes and human glioma cells, ACKR3 can be coupled to Gα-protein and triggers PLC and MAPK activation. GRK, G-protein receptor kinase; ADM, adrenomedullin; PAMP, proadrenomedullinN-terminal 20 peptide; PLC, phospholipase C; MAPK, mitogen-activated protein kinase. (Figure created with BioRender.com).

Because of its various ligands, ACKR3 can initiate downstream effects through several modes of action; in particular regarding the CXCL12/CXCR4/ACKR3-related signalling (21, 49, 50) (**Figure 1**). One of the first functions identified for ACKR3 is its scavenger activity where ACKR3 is internalized upon chemokine binding which leads to the lysosomal degradation of its ligands. Thus, ACKR3 regulates extracellular CXCL12 concentrations leading to modulation of CXCL12/CXCR4-mediated signaling (51–53). ACKR3 also acts as a scavenger receptor to regulate the migration of CXCR4 expressing cells (52).

Unlike many receptors, ACKR3 is not necessarily degraded after activation and internalization. ACKR3 continuously cycles between the plasma membrane and intracellular compartments (53) and can also regulate the expression of CXCR4 and CXCR4-mediated signalling. Cardiac stem cells (CSCs) express CXCR4 and ACKR3 and their expression and cooperation is necessary for the regulation of cell migration. ACKR3 inhibits CXCR4-dependent migration of CSCs (54). Furthermore, in cardiomyocyte derived from human induced pluripotent stem cells (hiPSCs), it has been shown that *Cxcr4* silencing reduced ACKR3 expression at the membrane suggesting a contribution of CXCR4 to the ACKR3 membrane trafficking. Although CXCR4 and ACKR3 downregulation resulted in a delayed cardiac phenotype, only knockdown of CXCR4 impaired the spontaneous beating of hiPSC-derived cardiomyocytes (55), suggesting that CXCR4 and ACKR3 govern differential effects during cardiac lineage differentiation. Interestingly, conditional deletion of *Ackr3* in interneurons regulates cortical neuron migration. In this model, ACKR3 controls CXCR4 protein levels that is made available for efficient signaling at the cell surface, thereby adapting chemokine responsiveness to migratory CXCR4-expressing cells (42, 43).

ACKR3 can also recruit and activate the β-arrestin signalling constitutively or after binding of its ligand (16, 32, 35) (56) (**Table 1**). CXCL12, but also CXCL11 and TC14012, a synthetic CXCR4 inverse agonist peptide and ACKR3 agonist, induce ß-arrestin 2 recruitment (16, 32, 57) and activate MAPK-associated signalling pathways (16, 35). β-arrestin 2 pathways are known to play an important role in cell survival and proliferation *via* ERK1/2 and Akt activation (16, 58). In addition, ACKR3 can modulate the signalling and functions of other GPCRs receptors or membrane proteins (**Table 1**). ACKR3 can modulate CXCL12 signalling either by regulating CXCR4 activity through ACKR3-CXCR4 heterodimers formation or by regulating the CXCL12 gradient through its scavenging activity (17, 36). Furthermore, it has also been shown that this heterodimer potentiates β-arrestin activation with increased stimulation of the ERK1/2 cascade and activation of p38, MAPK and SAPK (36, 38). Beyond interactions with cCKRs, ACKR3 is also able to interrelate with other membrane proteins. Indeed, ACKR3 can interact with Connexin-43 (Cx-43) in a CXCL12 dependent-manner. ACKR3 activation by CXCL12 promotes β-arrestin-2 and Cx-43 internalization which inhibits gap junctional intercellular communication in primary astrocytes (28). Cx-43 is a major protein of cardiac ventricular gap junctions and is crucial to cell-cell communication and cardiac function. Thus, this study suggests that ACKR3 and Cx-43 could interact in the heart, modulates cardiomyocyte phenotypes and thus cardiac function. However, the lack of tools such as molecules or synthetic peptides that prevent heterodimerisation does not allow confirmation of the existence of these interactions *in vivo*.

Adrenomedullin (ADM) is a peptide hormone critical for cardiac vascular and lymphatic development (59). ADM binds the GPCR Calcitonin receptor-Like Receptor (CLR) which can only be exported to the cell surface through its heterodimerization with one of the three Receptor Activity-Modify Proteins (RAMPs). A complex with either RAMP2 or RAMP3 confers a receptor selective for ADM and those with RAMP1 creates a receptor for calcitonin gene-related peptide (CGRP) (60)**.** Phenotypic analysis of *Ackr3* ^-/-^ and *Adm^-/-^* mice revealed that these mice exhibited similar cardiac and lymphatic vascular defects. Overexpressing of *Adm* in mice caused cardiac hypertrophy during embryogenesis similar to that observed in *Ackr3* ^-/-^ animals (61). Interestingly, *Ackr3* deficiency onto genetic background with *Adm* haploinsufficiency (*Adm^+/-^* mice) partially rescued the lethal defects in *Ackr3^-/-^* mice by preventing cardiac hyperplasia suggesting that ACKR3 scavenging activity is required for controlling ADM concentrations during development (46). Recently, RAMP3 was identified as an interacting partner of ACKR3 *in vitro* able to control its signalling and trafficking properties without affecting β-arrestin coupling in response to either AM or CXCL12 ligands. Thus, ACKR3-RAMP3 interactions scavenge ADM thereby reducing bioavailability of ADM and signaling mediated by CLR-RAMP3 receptor (62). Recent studies confirmed the interaction between ACKR3 and RAMP3 and the absence of impact of RAMP on ACKR3 activity (β-arrestin recruitment) in response to ADM. However, the ability of ACKR3-RAMP3 to scavenge ADM is lower than those of CLR/RAMP2 or CLR/RAMP3 receptors (30, 63). These data question the role of ACKR3 as ADM receptor. Further and more precise binding studies are needed before adding ADM to the ACKR3 ligand. Collectively, these studies suggest that the regulatory role of ACKR3 in the ADM signaling axis is either indirect, requires an accessory protein or occurs in a particular microenvironment.

Expression of ACKR3 in a wide variety of neuronal cells, vascular cells and immune cells as well as its up-regulation under hypoxic and inflammatory conditions, which are features of ischemic CVD, have made ACKR3 a central player in the regulation of homeostatic processes but also in inflammatory and ischemic diseases **(** **Table 2** **).**

**Table 2:** ACKR3 in cardiovascular diseases.

**Atheroprotective effects**	
Ubiquitous (CAG-Cre+*Cxcr7^flox^Apoe^−/−^*) and conditional *Ackr3^-/-^* (CAG-CreERTM *Cxcr7^flox^/flox Apoe_−/−_*) Increased serum cholesterol levels and hyperlipidemia-induced monocytosis	(64)
Hyperlipidemic *Apoe^-/-^* and *LdlR*-/- mice treated with agonist CCX771 *Reduced serum cholesterol, triglyceride levels and inhibited lesion formation after vascular injury*	(65)
Hyperlipidemic *Apoe^-/-^ Ackr3^-/-^* mice *Increased neointimal formation and lesional macrophage accumulation after vascular injury*	(65)
Endothelial *Ackr3^-/-^ (Cxcr7^flox/flox^ Cdh5-Cre)* *Increased neointimal formation and impaired re-endothelialization after vascular injury*	(64)
C57BL/6 mice treated with agonist VUF11207 *Reduced arterial thrombosis*	(66)
**Vasculogenesis**	
Conditional smooth muscle specific deficiency of *Cxcl12* (SM22CRE-*Cxcl12^flox/flox^*) *Inhibition of ACKR3 expression which induces severe vascular defects*	(67)
Endothelial Ackr3^-/-^ (Cxcr7^flox/flox^ Cdh5-Cre) in hind-limb ischemia mice model *Reduced blood flow recovery and vascular density in the ischemic gastrocnemius*	(64)
**Cardioprotective effects**	
Endothelial *Ackr3^-/-^ (Cxcr7^flox/flox^ Cdh5-Cre)* *Impaired cardiac function, reduced survival rate, increased infarct size and elevated plasma levels of CXCL12*	(64)
Cardiomyocytes *Ackr3^-/-^* (αMHC-Cre^+/-^ CXCR7^flox/flox^) *Excessive left ventricular dilatation and major systolic dysfunction after MI*	(68)
Fibroblasts *Ackr3^-/-^* (Col1a2-CreERT2^+/-^ CXCR7^flox/flox^) *No significant impact on heart function under basal condition or after MI*	(68)
Conditional smooth muscle specific deficiency of *Cxcl12* (SM22CRE-*Cxcl12^flox/flox^*) treated with agonist TC14012 *Reduced cardiac hypertrophy and attenuated vascular defects*	(67)
C57BL/6 mice overexpressing ACKR3 or treated with agonist TC14012 *Improved heart function, reduced infarct size and enhanced angiogenesis after MI*	(64, 69)
C57BL/6 mice treated with agonist VUF11207 before MI *Reduced thrombo-inflammatory response through modulation of the platelet lipidome, infarct size and improved cardiac function in a ischemia reperfusion mice model*	(66)
Silencing of Ackr3 (lentiviral shRNA) in C57BL/6 mice *Inhibition of M1 macrophages polarization and reduction of inflammation post-MI*	(70)
**Protective role in stroke**	
C57BL/6 mice injected with anti-ACKR3 neutralizing antibody *Enhanced neurogenesis in the hippocampal associated to cognitive functional recovery following cerebral ischemia*	(71)

## ACKR3 in Cardiovascular Diseases

### Cardiovascular Development

*Ackr3* deficient mice (*Ackr3^-/-^*) develop normally during early embryonic stages. At embryonic gestation days 18.5, animals die *in utero* or shortly after birth due to significant defects in the development of the cardiovascular system (38–40). Analysis of these animals showed cardiac hyperplasia associated with cardiomyocyte hypertrophy, aortic and pulmonary valve stenosis without mitral and tricuspid valve involvement, as well as atrial and ventricular septal defects, suggesting a major role of ACKR3 in cardiovascular development. Transcriptomic analyses also revealed a decrease in the expression of certain factors essential to vascular growth in semilunar valves (38). Of those that do survive to adulthood (30%), most animals have impaired heart function, cardiac hyperplasia, severe aortic valve calcification and thickening of aortic leaflets causing sudden death (40). These abnormalities were mainly associated to endothelial cell dysfunction because these defects were similar in endothelial cell specific *Ackr3* knockout mice (38). *Ackr3* has also been deleted in cardiomyocytes and fibroblasts but no changes were observed in cardiac phenotypes compared to control wild-type mice (68). More recently, conditional smooth muscle specific deficiency of *Cxcl12* (SM22CRE-*Cxcl12^flox/flox^*) revealed that CXCL12 was involved in cardiac and vascular homeostasis by regulating ACKR3 expression and its signalling. Indeed, in these mice, a high embryonic lethality characterized by developmental defects of the cardiovascular system was observed concomitant with alterations of coronary arteries structure (67). Adult mice developed cardiac hypertrophy and severe vascular defects associated with increased fibrosis, apoptosis and dilated arteries with thinner walls leading to impaired cardiac function. Transcriptomic data analysis of these hearts revealed the activation of genes involved in signalling pathways associated with hypertrophic cardiomyopathy, collagen synthesis and extracellular matrix reorganization. CXCL12-mediated signalling pathways such as Akt and ERK1/2 were also increased. Interestingly, this phenotype was associated with downregulation of ACKR3 expression in coronary artery endothelial cells, indicating an important role of ACKR3 in vessel maturation and cardiac remodelling. Furthermore, treatment of these mice with ACKR3 agonist TC14012 attenuated these defects and restored cardiac function with a marked activation of pERK signalling, suggesting that activation of pERK signalling pathways induced by the binding of TC14012 to ACKR3 prevented the progression of cardiac hypertrophy (67) (**Figure 1**).

### Atherosclerosis

**Figure 2:**
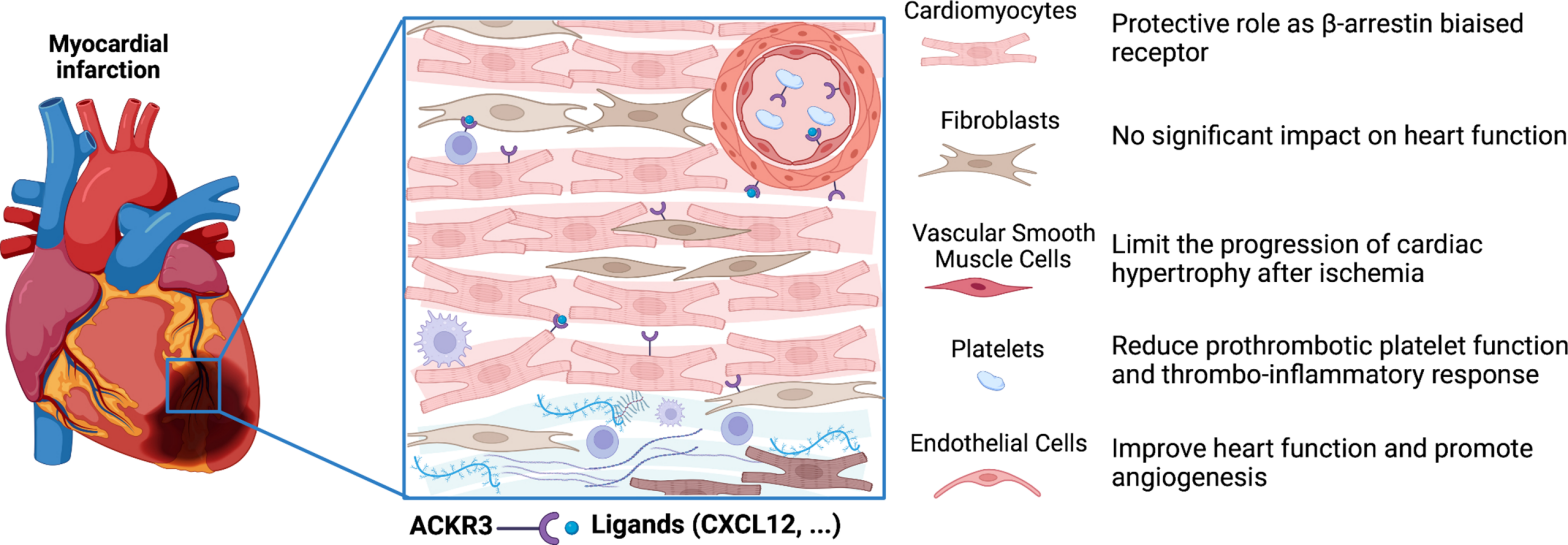
Role of ACKR3 in the infarcted heart. ACKR3 expression is increased in several cells of the heart following myocardial infarction, and can play multiple roles after activation by endogenous and exogenous ligands. ACKR3 exerts cardioprotective effects by acting either as a rheostat for certain ligands through scavenging activity or as a co-partner through dimerization with other receptors. In addition, ACKR3 may trigger activation of β-arrestin-dependent pathways. (Figure created with BioRender.com).

ACKR3 expression is significantly upregulated in macrophages and endothelial cells in aortic atheroma of hypercholesterolemic mice and human (64, 72, 73). Using atherosclerotic model of *ApoE-/-* mice, Li et al. demonstrated that ubiquitous and conditional knockout of *Ackr3* increased neointima formation and macrophage accumulation at lesion site after vascular injury. Administration of a pharmacological agonist of ACKR3 (CCX771) to *ApoE^-/-^* mice, inhibited lesion formation by reducing blood cholesterol through activation of its uptake within white adipose tissue. Thus, the athero-protective effects of ACKR3 are mainly mediated by the impaired cholesterol uptake within the adipose tissue (65). However, these findings also highlighted a cell specific functional response in particular in macrophages. Activation of ACKR3 by TC14012 in *ApoE-/-* mice fed a high-fat diet was also reported to limit atherosclerosis initiation by promoting endothelial repair and reducing atherosclerotic lesions through inhibition of pyroptosis signaling pathways. ACKR3 expression is significantly upregulated in both lesional and neointimal endothelial cells in murine and human arteries (64). Moreover, endothelial *Ackr3* deletion in mice also exacerbated neointimal hyperplasia following endothelial denudation injury, impaired re-endothelialization and promoted the development of atherosclerosis (64) (**Figure 2**).

ACKR3 is also constitutively expressed in human and murine platelets ant its expression is upregulated in patients with acute coronary syndrome (74, 75). Recently, ACKR3 activation, by the VUF11207 agonist, has been shown to exert anti-thrombotic actions through the modulation of the platelet lipidome (66). Indeed, its activation limited the generation of phospholipase C-derived lysophosphatidylcholine, a marker of plaque instability with distinct atherogenic properties. Thus, ACKR3 may regulate the generation of pro-thrombotic and pro-atherogenic lipids carried by platelets to the site of vascular injury **(** **Figure 2** **)**.

Although, these studies demonstrated an athero-protective role of ACKR3, some studies suggested potential detrimental effects. Indeed, ACKR3 expression was detected in macrophage in aortic atheroma of *ApoE^-/-^* mice but not in healthy aorta, suggesting that ACKR3 plays a role in inflammation at the lesion. In line with this reasoning, ACKR3 expression is also correlated with monocyte to macrophage differentiation as well as with the degree of maturation of B cells or that of dendritic cells (73, 76, 77). The differentiation of monocyte to macrophage is a key event in the formation of foam cells in atherosclerotic lesions. During this process, ACKR3 plays a crucial role in the regulation monocyte-macrophage function and induces pro-inflammatory signaling pathways in macrophages (73, 78). In addition, B cells regulate atherosclerotic plaque development through production of antibodies and cytokines. Thus, secretion of pro-inflammatory cytokines and antibodies influenced by ACKR3 may foster pro-atherosclerotic events. Future investigations are needed to understand whether the regulation of ACKR3 expression during this differentiation processes could contribute to the progression of atherosclerosis. Taken together, these findings demonstrate that ACKR3 has cell specific and process-specific functional outcomes.

### Stroke

CXCL12, CXCR4 and ACKR3 are widely expressed in the adult mouse and human brain (20, 79, 80) and plays a critical role in brain repair after ischemic stroke (81–84). Over-expression of CXCL12 by gene therapy shows protective effects and improves brain repair after ischemic injury (85). After focal ischemic stroke in mice, CXCL12, CXCR4 and ACKR3 expression is rapidly upregulated. In mice, CXCL12 and CXCR4 are strongly increased in the ischemic region and ACKR3 expression was largely expressed in the blood vessels in the peri-infarcted area, suggesting that ACKR3-dependent pathways may influence vascular and neuronal functions (86–88). In human, expression levels of both CXCL12 and ACKR3, but not that of CXCR4, are increased in the peri-infarction area of ischemic cerebral cortex, suggesting that CXCL12 modulates the repair of the brain after a stroke through ACKR3 activation (89). Stumm *et al.* showed a differential distribution of CXCL12 isoforms in the brain after focal cerebral ischemia suggesting an isoform-specific regulation of CXCL12 after injury (90). Indeed, CXCL12β isoform was selectively expressed by vascular endothelial cells and its upregulation was associated with a concomitant infiltration of CXCR4-expressing inflammatory cells whereas CXCL12α is expressed in neurons and modulates neuronal plasticity. Thus, one can speculate that isoform-specific regulation of CXCL12 may be due to the differential expression of ACKR3 and/or to the capacity of ACKR3 to scavenge CXCL12 and thereby modulate the CXCL12-dependent response. Recently, neutralization of ACKR3 with a specific blocking antibody following cerebral ischemia resulted in significantly enhanced neurogenesis in the hippocampal associated to cognitive functional recovery (71). These effects could be explained by the elevated levels of CXCL12 resulting from ACKR3 neutralization or by the modulation of CXCR4 signaling through CXCR4-ACKR3 heterodimers. However, further studies are needed to investigate the direct effect of ACKR3 on neurogenesis using knockout *Ackr3* mice models or by distinct RNA silencing of ACKR3.

### Myocardial Infarction

ACKR3 is the most highly expressed 7-transmembrane domain receptor in mouse heart (68). Single-cell RNA sequencing in mouse heart showed its expression in cardiomyocytes, fibroblasts, endothelial cells and smooth muscle cells (68). Expression of ACKR3 is significantly increased in the infarct border zone of mouse heart and in heart failure patients (68). Conditional endothelial *Ackr3* invalidation model (*Cxcr7^flox/flox^ Cdh5-Cre* model) showed excess mortality, impaired cardiac function associated with increased MI size, reduced angiogenesis and pro-fibrotic remodelling after experimental MI. The absence of ACKR3 could promote CXCR4-dependent signalling known to induce fibrosis. Conversely, cardiac overexpression of ACKR3 or ACKR3 agonist TC14012 treatment preserve cardiac function, enhance angiogenesis and reduce infarct size after MI in C57BL/6J mice (64, 69). These effects underlined the crucial role of endothelial ACKR3 in cardiac remodelling after MI. Activation of ACKR3 *in vitro* by the agonist TC14012 promotes proliferation and vascular tube formation of murine endothelial cells through ERK and PI3K/Akt pathways (64, 69, 91). In addition, ACKR3 is a critical regulator of mesenchymal stem cells-mediated postnatal vasculogenesis and arterial specification *in vitro* and *in vivo via* Notch signalling. These effects have been associated with the activation of ACKR3 expression on mesenchymal stem cells by VEGF and PDGF (41). Alternatively, several studies showed that ACKR3 governs the survival and proliferation of endothelial progenitor cells (EPCs) and their adhesion onto the endothelium contributing to angiogenesis (92, 93). The absence of ACKR3 in endothelial cells also decreases post-ischemic revascularization (64). In addition, EPC from diabetic mice showed decreased expression of ACKR3 and reduced capacity for vascular tube formation. CXCL12/ACKR3 interactions allow EPCs to better resist oxidative stress and improve their angiogenic potential in the context of diabetes-related lower limb ischemia (92).

Cardioprotective effects of ACKR3 could also be mediated by its capacity to impact platelet-related activities. Expression of ACKR3 in platelets is increased post-MI in mice and patients with acute coronary syndrome (94) and is associated with improved cardiac function and prognosis (78, 94). Pro-survival and anti-thrombotic effects mediated through the binding of MIF to ACKR3 have been demonstrated *in vivo* in murine model of atherothrombosis (95). Administration of specific and selective ACKR3 agonist, VUF11207, reduced thrombo-inflammatory response, infarct size and improved cardiac function in a ischemia-reperfusion mice model (66). In these mice, reduced platelet-leukocyte aggregation in peripheral circulation and lower plasma levels of several inflammatory mediators were observed supporting the cardioprotective role of platelet ACKR3. Interestingly, treatment with ACKR3 agonist does not affect basal hemostatic or coagulation response. Thus, the anti-thrombotic actions of ACKR3 through the modulation of the platelet lipidome to limit metabolism and release of thrombotic and atherogenics mediators strengthened the therapeutic potential of ACKR3 in CVD (96). However, the mechanisms underlying this anti-thrombotic action of ACKR3 in platelets remain ascertained and further studies are needed.

ACKR3 expression is increased in cardiomyocytes of patients with heart failure (68). Genomic analysis of single-cardiomyocyte RNA-seq data from human heart biopsies of pre- and post-implanted patients with left ventricular assist device revealed that implantation normalized this *Ackr3* upregulation in both ischemic and non-ischemic cardiomyopathies (97) indicating a crucial role of ACKR3 in human cardiomyocytes. Interestingly, conditional specific deletion of *Ackr3* in cardiomyocytes (*αMHC-Cre^+/−^ CXCR7^flox/flox^* model*)* in mice leads to excessive left ventricular dilatation and major systolic dysfunction after MI demonstrating that cardiomyocyte ACKR3 protects the heart after ischemia (68). This cardioprotective effect of ACKR3 involves ERK activation induced by β-arrestin signalling in cardiomyocytes. In agreement with this result, the expression of ACKR3 is significantly upregulated concomitantly with ERK activation at the border zone of infarcted heart. These results indicate that ACKR3 may play a cardioprotective role by acting as a β-arrestin-biased receptor. Therapeutic targeting of β-arrestin-biased signalling through activation of β-adrenergic (carvediol) (98) and angiotensin II receptors (TRV120067) (99) showed cardioprotective effect in pre-clinical models. Collectively, these data support the fact that β-arrestin-biased signalling mediated by ACKR3 may be a new target in the treatment of CVD. Although ACKR3 is also expressed in cardiac fibroblasts, which are involved in cardiac repair after MI, no impact on cardiac function was observed in infarcted mice with conditional ACKR3 deletion in fibroblasts (*Col1a2-CreERT2* x *Cxcr7^flox/flox^* model) (68) (**Figure 1**).

Cells of the immune system have been shown to mediate both protective and damaging effects in heart remodeling (100–102). ACKR3 is expressed in inflammatory cells (103) and its expression is especially correlated with monocyte to macrophage differentiation (73), the degree of maturation of B cells (76) or that of dendritic cells (77). ACKR3 expression in macrophages is upregulated during polarization *in vitro* and following MI with positive correlation with M1 but not M2 (70, 73) like macrophage markers. In these macrophages, ACKR3 controls CXCL12 and CXCL11-mediated chemotaxis as well as the production of pro-inflammatory cytokines such as IL-1 and Il-6 after MI indicating a deleterious role of ACKR3 in macrophages in this setting. Invalidation of *Ackr3* by shRNA before induction of MI inhibited macrophage polarization, chemotaxis and inflammation and reduced infarct size leading to an improvement of cardiac function post-MI (70). Unfortunately, the role of ACKR3 in different immune cell sub-populations in post-MI cardiac remodeling are incomplete. Hence, studies investigating the specific role of ACKR3 in each immune cell type would allow to better understand its role in CVD.

## Conclusions and Future Directions

Various pharmacological tools targeting ACKR3 have been tested in murine model of atherosclerosis, myocardial infarction, stroke and different knockout murine models for ACKR3 have been developed to highlight its role crucial in CVD. However, although these studies clearly demonstrated that ACKR3 may elicit a cell- and tissue-specific functional responses, the molecular mechanisms controlling ACKR3-related functions in CVD are a topic still debated. Tissue-dependent functioning can be related to the capacity of ACKR3 to interact with numerous ligands and can depend on surrounding cells producing and processing these ligands. Up-regulation of ACKR3 expression and those of its ligands in response to hypoxia, inflammation or infection suggests that ACKR3 function may vary under these different pathologic conditions. The fact that ACKR3 heterodimerizes with other proteins or that ACKR3 signaling can interfere with numerous intracellular pathways add another level of complexity. One main issue will be to analyze the functional consequences of these interactions ligand or protein/ACKR3 to identify new potential targets. As mentioned in this review, an unique function cannot be assigned to ACKR3. Therefore, the molecular mechanisms underlying distinct signaling pathways and functional responses downstream of ACKR3 need to be further investigated to better understand ACKR3 functions, and develop tailored therapeutics for CVD.

## Author Contributions

VD and AL wrote the initial manuscript draft. PA conceived the Table and Figure. AL and J-SS edited the draft and with all other authors contributed to editing manuscript and approved the submitted version.

## Funding

This work was supported by Inserm, Université Paris Cité. PA and VD were supported by the "Fondation pour la Recherche Médicale".

## Conflict of Interest

The authors declare that the research was conducted in the absence of any commercial or financial relationships that could be construed as a potential conflict of interest.

## Publisher’s Note

All claims expressed in this article are solely those of the authors and do not necessarily represent those of their affiliated organizations, or those of the publisher, the editors and the reviewers. Any product that may be evaluated in this article, or claim that may be made by its manufacturer, is not guaranteed or endorsed by the publisher.

## Abbreviations

ACKR3, atypical chemokine receptor 3; ADM, adrenomedullin; cCKR, conventional chemokine receptor; CGRP, calcitonin gene-related peptide; CVD, **c**ardiovascular diseases; CXCL11, C-X-C motif chemokine ligand 11; CXCL12, C-X-C motif chemokine ligand 12; CXCR7, C-X-C motif chemokine receptor 7; GPCR, G-protein-coupled receptor; MIF, macrophage migration inhibitory factor; PAMP, proadrenomedullin N-terminal 20 peptide; SDF-1, stromal derived factor-1; RAMP, receptor activity-modify proteins.
